# Evaluating antibiosis resistance to cabbage aphid (*Brevicoryne brassicae* L., 1758) in vegetable brassicas (*Brassica oleracea* L.) and related C‐genome *brassica* species

**DOI:** 10.1002/ps.70161

**Published:** 2025-08-29

**Authors:** Andrew K Gladman, Gill Prince, Graham Teakle, David Chandler

**Affiliations:** ^1^ Warwick Crop Centre, School of Life Sciences University of Warwick Warwick UK

**Keywords:** host plant resistance, partial resistance, varietal resistance, integrated pest management, *Brassica cretica*, *Brassica villosa*

## Abstract

**BACKGROUND:**

Nineteen *Brassica* accessions from four C‐genome species were screened under controlled environment conditions for their effects on the development of the cabbage aphid, *Brevicoryne brassicae*. The aim was to identify accessions with antibiosis host plant resistance (HPR) to *B. brassicae*. Candidate *Brassica* accessions were selected because of (i) upregulation of transcription factors gene orthologues acting within the jasmonic acid signalling pathway, or (ii) having been reported in the 1990s as partially resistant to cabbage aphid. Accessions were evaluated by placing three 1‐day‐old aphid nymphs on six true leaf stage plants and recording the aphid population size 14 days later.

**RESULTS:**

There was significant (GLM, *P* < 0.05) variation in aphid population size between the accessions. Aphid populations on the most resistant accessions were approximately one third the size of those on the most susceptible accessions. Two accessions (*B. cretica*, *B. villosa*) supported significantly fewer aphids compared to a commercial variety of *B. oleracea* used as a reference control. Eight accessions (including *B. cretica*, *B. oleracea* and *B. villosa*) were selected for further study of varietal effects upon aphid biology. Significant differences in *B. brassicae* reproduction, intrinsic rate of increase, and population doubling time were identified between these eight accessions.

**CONCLUSION:**

Linear regression analysis identified that HPR was explained mainly by varietal effects on aphid pre‐reproductive period, which accounted for 53.9% of the variation in population development resistance screening results. Partial HPR could be a valuable trait for plant breeding to limit aphid population development as part of an integrated pest management strategy. © 2025 The Author(s). *Pest Management Science* published by John Wiley & Sons Ltd on behalf of Society of Chemical Industry.

## Graphical abstract

Nineteen *Brassica* accessions were screened for antibiosis resistance to *Brevicoryne brassicae*. Significant variation in resistance was identified. Detailed bioassays suggest that varietal effects upon aphid pre‐reproductive period conferred observed resistance.

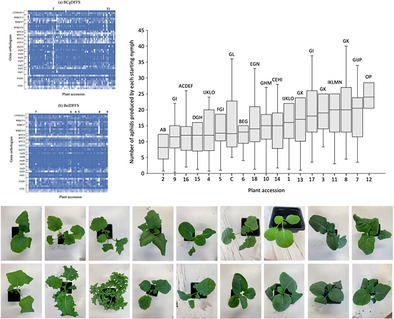

## 1 INTRODUCTION

Global production of horticultural (fruit and vegetable) crops is significantly constrained by insect pests which affect both yield and crop quality, and there is a need for more effective, sustainable methods of crop protection. Most experts agree that this is best achieved using integrated pest management (IPM).^1^, ^2^ IPM is a systems approach in which different plant protection tools are combined in complementary ways to reduce and maintain pest populations below their economic damage threshold while minimizing applications of synthetic chemical pesticides.^3^

Heritable host plant resistance (HPR) to insect pests should be a core component of IPM because of its direct effects upon pest populations and its potential to interact synergistically with other IPM tools.^4^, ^5^, ^6^ To date, >500 commercial crop cultivars have been bred with HPR to one or more invertebrate pest species, predominantly in arable crops, including maize, wheat, soybean and rice.^7^ Far less attention has been paid to breeding horticultural crop cultivars with HPR,^7^, ^8^ possibly because of the high quality thresholds in horticultural crops and the hitherto ready availability of low cost and highly effective synthetic chemical pesticides (Ellis PR, 2020, pers. comm.).^6^ However, the evolution of insecticide resistance in target pest populations, together with government restrictions on pesticides to protect human and environment safety^9^, ^10^ has meant that restoring heritable HPR in modern horticultural crop varieties is becoming increasingly important.^4^, ^6^ Horticultural wild crop ancestors and land races are considered to be a valuable source of pest resistance genes, many of which have been unintentionally lost during the breeding of modern crop varieties.^4^, ^6^ Therefore, screening germplasm collections should be a useful way of rediscovering these ‘lost’ genes for plant breeding programmes.

A majority of HPR research to date has focused on identifying complete resistance encoded by dominant R‐genes.^11^ Complete HPR is, understandably, attractive for seed companies, but it is rare.^12^ Where it does occur and is bred into modern varieties, there is a high risk of breakdown owing to the strong selection pressure on pest populations to evolve countermeasures.^12^, ^13^, ^14^, ^15^ An alternative approach is to utilize partial HPR bred into the crop as a quantitative, multigenic trait. Partial HPR can act by reducing a pest's rate of development or reproduction, thereby limiting pest population growth.^12^, ^16^ Partial HPR is relatively common within plant gene pools, but because it is conferred through the action of multiple ‘minor effect’ genes, it poses a greater challenge for breeders to incorporate into elite varieties. However, once in place, it is likely to be durable, because the probability of a pest genotype evolving countermeasures to each constituent of a suite of minor effect genes should be lower compared to a single dominant gene exerting a large selective force.^11^, ^17^ For this reason, partial HPR is increasingly being recognized as a valuable tool for integration into IPM strategies.^14^, ^18^

Complete and partial HPR to insect pests can arise through a range of physical and molecular mechanisms. Physical resistance traits include increased leaf thickness, leaf surface waxes, and the presence of trichomes, both glandular and nonglandular.^4^ Molecular mechanisms of resistance meanwhile include phloem‐plugging proteins, reduced phloem sap nutrient content, and the production of toxic or unpalatable secondary metabolites.^4^, ^12^ Despite the evolutionary importance of physical resistance traits, to date a majority of plant breeding efforts have focussed primarily upon molecular mechanisms of resistance to invertebrate pests.^4^

Molecular mechanisms of resistance can be expressed constitutively or induced in response to infestation.^4^, ^12^ Inducible resistance can be activated either by detection of generalized aphid‐induced tissue damage or gene‐for‐gene recognition of aphid‐secreted effector molecules by plant resistance genes (R‐genes).^12^ Successful recognition of aphid effectors triggers signalling cascades leading to upregulation of defence‐related genes.^12^ Research suggests that feeding by specialist aphids such as the Brassica‐specialist cabbage aphid (*Brevicoryne brassicae*) causes local activation of both the jasmonic acid (JA) and salicylic acid (SA) signalling pathways – the two phytohormonal pathways most frequently associated with HPR against aphid pests.^19^, ^20^ Recent reviews highlight that SA signalling is more commonly detected in response to sucking pests whereas JA signalling is more commonly activated in response chewing pests;^21^ however, both pathways appear to play a significant positive role in the specific case of HPR to aphid pests.^22^ The relative importance of the JA and SA pathways in HPR to aphids appears to vary dependent upon the specific ‘plant × aphid’ situation evaluated, with significant evidence presented since the mid‐2000s demonstrating conflicting results between crop species.^23^, ^24^, ^25^, ^26^, ^27^, ^28^, ^29^, ^30^, ^31^, ^32^ Many authors also highlight the significant interaction between the JA and SA pathways, which results in antagonistic and/or interdependent interplay in their effects upon aphid infestation, again varying between plant and aphid species.^21^, ^27^, ^31^, ^33^

In this paper, we evaluated accessions of *Brassica oleracea* and related C‐genome *Brassica* species for their antibiosis resistance to cabbage aphid, *B. brassicae*. Globally, aphids (Hemiptera: Aphididae) are among the most significant insect pests of *Brassica* crops.^34^, ^35^, ^36^ They are characterized by short development times, parthenogenetic reproduction and high fecundity, meaning that, on susceptible host plants, aphid populations increase rapidly, leading to significant economic damage where uncontrolled.^37^

Cabbage aphid, *B. brassicae*, is a major pest of *Brassica* crops worldwide.^37^, ^38^, ^39^ It can cause significant reductions in marketable yield from direct feeding and cosmetic damage. *Brevicoryne brassicae* also is a vector of range of damaging plant pathogenic viruses including turnip mosaic virus (TuMV) and cauliflower mosaic virus (CaMV).^37^, ^38^ It exhibits a holocyclic life cycle, overwintering as eggs on *Brassica* plants which emerge in favourable spring conditions, although in the UK and other temperate areas a majority of *B. brassicae* populations now persist year‐round as viviparous females on cultivated *Brassica* crops, agricultural escapees (volunteers), Brassicaceae weeds and wild species relatives.^34^, ^37^, ^40^

Both historically and presently, the control of *B. brassicae* has relied predominantly on routine applications of synthetic chemical pesticides, with current options available to UK growers including cyantraniliprole, deltamethrin, flonicamid, lambda‐cyhalothrin and spirotetramat.^41^ While insecticide resistance in *B. brassicae* has yet to be reported in Europe, there is evidence for pesticide insensitivity and resistance in *B. brassicae* populations in Pakistan.^42^

We evaluated *Brassica* accessions from four different C‐genome species (*Brassica cretica*, *B*. *macrocarpa, B. oleracea*, *B. villosa*) for their effects on *B. brassicae* population development under controlled environment conditions. This work builds on research from the 1990s, which identified partial antibiosis HPR in vegetable Brassica wild species relatives.^43^, ^44^, ^45^, ^46^, ^47^, ^48^ We also included a set of *B. oleracea* accessions selected on the basis of upregulated expression (mRNA sequence reads) of transcription factor orthologues active at different stages in the *Arabidopsis thaliana* JA signalling pathway (COI1, PAD4, JAZ1‐12, MYC2‐4, WRKY7 WRKY11 WRKY 44 and CYP81D11).^21^, ^25^, ^26^, ^49^, ^50^ The JA pathway was selected as the phytohormone signalling pathway of focus owing to strong evidence of both (1) constitutive JA pathway upregulation leading to improved *Brassica* HPR to *B. brassicae* and (2) JA nonexpressing mutants demonstrating significantly elevated susceptibility to *B. brassicae* infestation.^25^, ^26^

## 2 MATERIALS AND METHODS

### 2.1 Biological materials

#### 2.1.1 Brevicoryne brassicae

The *B. brassicae* biotype used in this study was the K3 clone isolated from Brussels sprouts in Lincolnshire, UK (1997). This *B. brassicae* clone demonstrates no known pesticide resistance. *Brevicoryne brassicae* cultures were maintained on 5‐week‐old Brussels sprouts plants, *B. oleracea* var. *Gemmifera* ‘Doric F1’ within mesh cages (50 × 50 × 50 cm Bugdorm‐6S610; Watkins & Doncaster, Leominster, UK) in a controlled environment room ([20 ± 2 °C], 60% relative humidity (RH), 16 h:8 h, light:dark, fluorescent tube lighting). Every 2 weeks, new plants were added to the aphid colony and older plants discarded. Fixed‐age *B. brassicae* cultures were produced by transferring groups of 20 mature apterous virginoparae onto 5‐week‐old *B. oleracea* var. *Gemmifera* ‘Doric F1’ plants. After 24 h all mature virginoparae were removed and the fixed‐age progeny were maintained for a further nine days for use in experiments.

#### 2.1.2 *Brassica* plants – growing and candidate accessions selection

*Brassica* seeds were sown in clear plastic boxes (15 × 6 cm) filled with moist vermiculite (BHGS, Evesham, Worcestershire, UK) and maintained in a controlled environment growing room at 20 ± 2 °C and 16 h:8 h, light:dark photoperiod. After 7 days, germinated seedlings were transplanted into Levington M2 compost in 75 mm black polyethylene pots and grown on for 4 weeks (20 ± 2 °C, 60% RH, 16 h:8 h, light:dark photoperiod), watering as required.

We selected 18 candidate *Brassica* accessions to test against *B. brassicae* (Table 1). A nineteenth *Brassica* accession also was included as a commercially representative reference control ‐ *Brassica oleracea* var. *Gemmifera* ‘Doric F1’. This control was selected as a widely grown commercial variety against which all other varieties could be compared. It was unknown at trial outset whether ‘Doric F1’ was significantly resistant or susceptible to *B. brassicae*.

**Table 1:** *Brassica* accessions selected for antibiosis resistance screening against cabbage aphid, *Brevicoryne brassicae*

No.	Selection criteria	Genus species	Subsp.	Kew cultivar group	Crop type	Cultivar
1	BCgDFFS	*Brassica macrocarpa*	—	—	—	—
2	BCgDFFS	*Brassica villosa*	*tinei*	—	—	—
3	BCgDFFS	*Brassica macrocarpa*	—	—	—	—
4	BolDFFS	*Brassica oleracea*	—	Gongylodes	Kohlrabi	—
5	BolDFFS	*Brassica oleracea*	—	Tronchuda	Tronchuda	—
6	BolDFFS	*Brassica oleracea*	—	Botrytis	Romanesco	—
7	BolDFFS	*Brassica oleracea*	—	Gemmifera	Brussels sprouts	—
8	BolDFFS	*Brassica oleracea*	—	Italica	Broccoli	—
9	BolDFFS	*Brassica oleracea*	—	Alboglabra	Chinese Kale	—
10	Ellis *et al*.^45^	*Brassica oleracea*	—	Acephala	Kale	‘Butzo’
11	Ellis *et al*.^45^	*Brassica oleracea*	—	Acephala	Kale	‘Furchehnkohl’
12	Ellis *et al*.^45^	*Brassica oleracea*	—	Acephala	Kale	‘Arsis F1’
13	Ellis *et al*.^45^	*Brassica oleracea*	—	Botrytis	Cauliflower	‘Tasman’
14	Ellis *et al*.^45^	*Brassica oleracea*	—	Botrytis	Cauliflower	‘Mikado March’
15	Ellis *et al*.^45^	*Brassica oleracea*	—	Botrytis	Cauliflower	‘Marzatico Napoletano’
16	Ellis *et al*.	*Brassica cretica*	—	—	—	—
17	Ellis *et al*.	*Brassica cretica*	—	—	—	—
18	Ellis *et al*.	*Brassica cretica*	—	—	—	—
‐	Reference Control	*Brassica oleracea*	—	Gemmifera	Brussels sprouts	‘Doric’

Nine of the accessions (accessions 9–18) were selected on the basis of having been reported previously as exhibiting antibiosis partial resistance to *B. brassicae* in two glasshouse tests.^45^ The remaining nine candidate accessions (accession nos. 1–9) were sourced from two Diversity Fixed Foundation Sets (DFFS) of *Brassica* genotypes developed and maintained by the DEFRA‐funded Vegetable Genetic Improvement Network project (VeGIN) at the University of Warwick Crop Centre, Wellesbourne (https://warwick.ac.uk/fac/sci/lifesci/research/grc/plant/dffs/)^51^ and the UK Vegetable Genebank (UKVGB; www.warwick.ac.uk/fac/sci/lifesci/wcc/genebank/).

DFFS consist of genetically fixed lines selected to encompass as broad a sample of diversity across the species/crop‐type gene pool as possible. Two DFFS collections were used as germplasm sources in this study:

- **BolDFFS:** a DFFS of 188 crop‐type *B. oleracea* accessions for which mRNAseq data were available for 50 accessions through the DEFRA‐funded Vegetable Genetic Improvement Network project (VeGIN) (https://warwick.ac.uk/fac/sci/lifesci/research/vegin/brassica/boldffs/)
- **BCgDFFS:** a DFFS of 89 accessions representing 14 C‐genome *Brassica* wild species for which mRNAseq data were available for 77 accessions through the DEFRA‐funded Vegetable Genetic Improvement Network project (VeGIN) (https://warwick.ac.uk/fac/sci/lifesci/research/vegin/brassica/bcgdffs/).^51^

Rather than selecting and screening a subset of *Brassica* accessions at random from each DFFS, a novel pre‐selection approach was trialled. This approach was developed on the basis of (1) the availability of a pre‐infestation transcriptomic data for a majority of accessions in both BolDFFS and BCgDFFS, and (2) published literature indicating that constitutive upregulation of jasmonic acid pathway genes may confer *Brassica* HPR to *B. brassicae*.^25^, ^26^ Through literature sources, 19 genes from the *Arabidopsis thaliana* JA signalling pathway were identified as targets to evaluate for relative gene expression levels. The 19 genes selected were predominantly transcription factors involved in signal recognition, signal transduction and transcriptional regulation at multiple stages of the JA signalling pathway.^25^, ^26^, ^49^, ^50^, ^52^

Orthologues of these 19 genes were identified in *B. oleracea* using polypeptide sequence reciprocal best Blast searches (www.blast.ncbi.nlm.nih.gov/Blast.cgi).^53^, ^54^ An identity match threshold of 60% was adopted to reduce type II errors owing to *A. thaliana* and the *Brassica* species being related at the family level (Brassicaceae).

Gene expression data (mRNA reads) generated during the DEFRA‐funded VeGIN project (available by request: https://warwick.ac.uk/fac/sci/lifesci/research/vegin/brassica/boldffs/) was extracted for each of the identified *Brassica* JA‐pathway gene orthologues for all accessions in BolDFFS and BCgDFFS. Using these gene expression data, a heatmap of relative gene expression levels was then generated for each DFFS (R Foundation for Statistical Computing, Vienna, Austria). Grand means of mRNA reads for each of the *Brassica* accessions also were calculated across all gene orthologues. Using the calculated grand means and the generated heatmaps, nine accessions (six from BolDFFS, three from BCgDFFS) which demonstrated the highest expression levels across all gene orthologues were selected for inclusion in aphid resistance screening. Where seed stocks were low for selected varieties, the next highest scoring variety was selected.

Owing to (i) the lack of genome sequences for most non‐*B. oleracea* wild C‐genome *Brassica* species included within BCgDFFS and (ii) the close relatedness of wild C‐genome *Brassica* species with *B. oleracea*, the same JA‐pathway gene orthologues identified for *B. oleracea* also were used for other C‐genome *Brassica* species.

### 2.2 Antibiosis resistance screen

Antibiosis resistance testing was undertaken on 5‐week‐old plants (6‐true leaf stage). Thirty plants of each of the 18 accessions were evaluated for resistance to *B. brassicae* using a randomized incomplete block design (nine candidate accessions + reference control included in each incomplete block; 10 plants per accession per block; six blocks in total; each experimental block undertaken on a separate occasion). Three fixed‐age, 10‐day old apterous virginoparae were transferred to the fourth true leaf of each plant, enclosed within a 5‐cm‐diameter clip cage, and left for 24 h to reproduce, after which the clip cage was removed along with all *B. brassicae* adults and all but three fixed‐age *B. brassicae* nymphs. Each plant with three 1‐day‐old nymphs was then sealed in a porous bread‐bag (200 × 250 mm perforated polypropylene bags; Cater4you Ltd, High Wycombe, UK) and maintained in a controlled growth room (20 ± 2 °C, 60% RH, 16 h:8 h, light:dark photoperiod) for 14 days, watering as required. Plants were then removed from bread bags and the number of aphid adults and nymphs per plant was counted.

### 2.3 Aphid development rate and reproduction

A subset of nine Brassica accessions were chosen for further study. A clip cage experiment was done to assess (1) the duration of each *B. brassicae* instar phase, (2) the total time taken from birth to adulthood (pre‐reproductive period), and (3) reproduction of *B. brassicae* upon reaching adulthood. Three *Brassica* plants of each *Brassica* accession were raised to 5 weeks old. Three fixed‐age, 10‐day‐old apterous virginoparae were then transferred to the fourth true leaf of each of the three plants per accession and left for 24 h to reproduce, after which all *B. brassicae* adults and all but one *B. brassicae* nymph were removed. The single nymph was then enclosed within a 5‐cm diameter clip‐cage *in situ* on the leaf. Plants were maintained at 20 ± 2 °C, 60% RH and 16 h:8 h, light:dark photoperiod for 20 days and watered as required. Every 24 h the clip cage was opened and inspected for the presence of a moulted cuticle, indicating progression to the next instar phase (ecdysis). Upon aphids reaching adulthood, the number of nymphs produced each day was recorded for a duration matching the identified pre‐reproductive period. All nymphs were removed each day after counting. This experiment was repeated on three separate occasions; thus, nine plants were assessed per *Brassica* accession.

Intrinsic rate of population increase (*r*_m_) was calculated according to the method described by Wyatt and White (1977),^55^ where *d* is the duration of the aphid pre‐reproductive period and *M*_*d*_ is the number of female offspring produced per original asexual aphid female from the first to the *d* ^th^ day of reproduction:

$$r_{m}=0.74\;\left(\mathrm{Ln}M_{d}\right)/d$$

Aphid population doubling time (*DT*) also was calculated as:^55^, ^56^, ^57^

$$\mathrm{DT}=\text{Ln2}/r_{m}$$

### 2.4 Aphid weight gain during development

Aphid mean relative growth rate was measured as follows: plants were grown to 5‐weeks old (6‐true leaf stage; 20 ± 2 °C, 60% RH, 16 h:8 h, light:dark photoperiod), then 20 10‐day‐old fixed‐age adult *B. brassicae* apterae were transferred to each plant for 24 h to reproduce, after which all adults were removed, leaving a cohort of 1^st^‐instar nymphs on each plant. Five nymphs per plant were then sampled destructively 1, 4, 7 and 10 days after removal of adults. Aphids were weighed, as a group of five aphids, using an Oahus balance. After weighing aphids on Day (D)10, all newly produced *B. brassicae* nymphs were removed, and remaining adult *B. brassicae* were left for 24 h to reproduce, generating a new generation of fixed‐age 1‐day‐old nymphs. All *B. brassicae* adults were then removed, leaving only the second generation of fixed‐age 1‐day‐old nymphs on plants. The weighing process used for the first generation of nymphs was then repeated on this second generation, again weighing five nymphs destructively on D1, D4, D7 and D10. Following weighing on D10, the process of generating a new fixed‐age population of 1‐day‐old nymphs was repeated a further time to produce a third generation of aphid nymphs, which were again weighed after 1, 4, 7 and 10 days, after which the experiment was concluded. The whole experiment (including all three aphid generations) was repeated on three occasions, with three replicate plants of each accession tested during each repeat of the experiment. Aphid weight over three aphid generations was therefore assessed on nine plants of each accession.

Mean relative growth rate (*MRGR*) for *B. brassicae* in each generation was calculated using weights (*W*_1_ and *W*_2_) at D1 (*t*_1_) and D7(*t*_2_).^58^ Days 1–7 were selected for calculation of MRGR due to weight increase having been linear between these days, with the rate of *B. brassicae* nymph weight increase observed to slow notably between D7 and D10.

$$\text{MRGR}=\left(\mathrm{Ln}W_{2}–\mathrm{Ln}W_{1}\right)/\left(t_{2}–t_{1}\right)$$

### 2.5 Aphid life‐history table and relationship between life parameters and initial partial resistance screening results

Having evaluated *B. brassicae* development rate to adulthood, rate of reproduction, maximum weight reached and mean relative growth rate on nine different *Brassica* accessions, a life‐history table was produced to summarize all data collected for each *Brassica* accession.

In order to evaluate which specific HPR effects accounted for the most significant proportion of variation in the initial antibiosis screening results, linear regression analysis was undertaken. Initial antibiosis screening results were included in this analysis as the dependent variable, with each measured *B. brassicae* biological metric included as the dependent variable in a stepwise fashion.

### 2.6 Statistical analysis

All statistical tests for both the initial population development antibiosis screen and subsequent in‐depth bioassays were performed using IBM Spss statistics for Macintosh, v27.0 (IBM Corp., Armonk, NY, USA). Data on aphid development time was transformed via a square‐root transformation and analyzed through ANOVA. All other datasets were not normally distributed; thus, analyses were undertaken using generalized linear modelling (GLM) or generalized linear mixed modelling (GLMM) including replicate as a random factor. All GLM and GLMM analyses used a negative binomial distribution, which best modelled the left‐skewed datasets. A custom dispersion parameter also was used, correcting for the presence of any overdispersion within datasets.

Four analyses were undertaken to evaluate the population development‐based antibiosis screen results; therefore, a Bonferroni correction was applied to account for these multiple analyses, altering significance thresholds to 0.0125. These four analyses were undertaken to determine whether the following were significant factors influencing *B. brassicae* performance: (1) Plant accession, (2) Plant accession selection criteria, (3) *Brassica* species and (4) *B. oleracea* cultivar group.

## 3 RESULTS

### 3.1 Candidate *brassica* accession selection – DFFS selected accessions

Through reciprocal best Blast searches, 49 gene orthologues of the 19 *A. thaliana* JA pathway transcription factor genes were identified in *B. oleracea* (Table 2). Of these 49 genes, mRNA sequencing read data were available for 43 genes for accessions in BolDFFS and BCgDFFS.

**Table 2:** Nineteen selected *Arabidopsis thaliana* genes acting as transcription factors within the jasmonic acid (JA) signalling pathway and identified *B. oleracea* gene orthologues

JA pathway gene	*Arabidopsis thaliana* gene genomic location	Identified *Brassica oleracea* orthologue genomic location	Percentage identity match
COI1	AT2G39940	Bo3g034570	89
		Bo4g025750	89
		Bo4g188660	78
		Bo4g188680	78
PAD4	AT3G52430	Bo7g102600	65
		Bo8g080690	66
		Bo4g125400	71
		Bo8g080720	68
JAZ1	AT1G19180	Bo5g027170	74
		Bo8g068340	73
		Bo8g102890	73
JAZ2	AT1G74950	*Bo6g119140*	*65*
		*Bo6g086720*	*67*
		Bo2g082410	69
JAZ3	AT3G17860	Bo1g119100	83
		Bo5g121480	80
JAZ5	AT1G17380	Bo5g023780	68
		Bo8g067140	68
		Bo8g104890	68
JAZ6	AT1G72450	Bo2g076750	73
		*Bo6g115920*	*71*
JAZ7	AT2G34600	*Bo4g182750*	*66*
JAZ8	AT1G30135	Bo3g147640	77
		Bo3g147730	73
		Bo5g062450	69
JAZ9	AT1G70700	Bo2g068460	73
		*Bo6g112300*	*67*
JAZ10	AT5G13220	Bo9g168900	78
		Bo2g011150	74
		Bo3g009050	74
JAZ12	AT5G20900	Bo2g023250	63
		Bo9g150390	67
MYC2	AT1G32640	Bo5g086990	87
		Bo8g028850	79
MYC3	AT5G46760	Bo7g075710	76
		Bo9g065530	74
MYC4	AT4G17880	Bo1g017990	73
WRKY33	AT2G38470	Bo3g032500	78
		Bo4g027280	75
		Bo4g187630	75
		Bo7g093150	65
WRKY7	AT4G24240	Bo1g037740	75
		Bo7g109430	74
WRKY11	AT4G31550	Bo1g011200	80
		Bo3g173880	79
		Bo7g115250	76
CYP81D11	AT3G28740	Bo2g146400	82
		*Bo7g042981*	*79*
		Bo9g008590	84

Transcriptomic data (mRNA sequencing reads) were unavailable for genes in italics, thus these genes were excluded from subsequent analyses.

**Figure 1:**
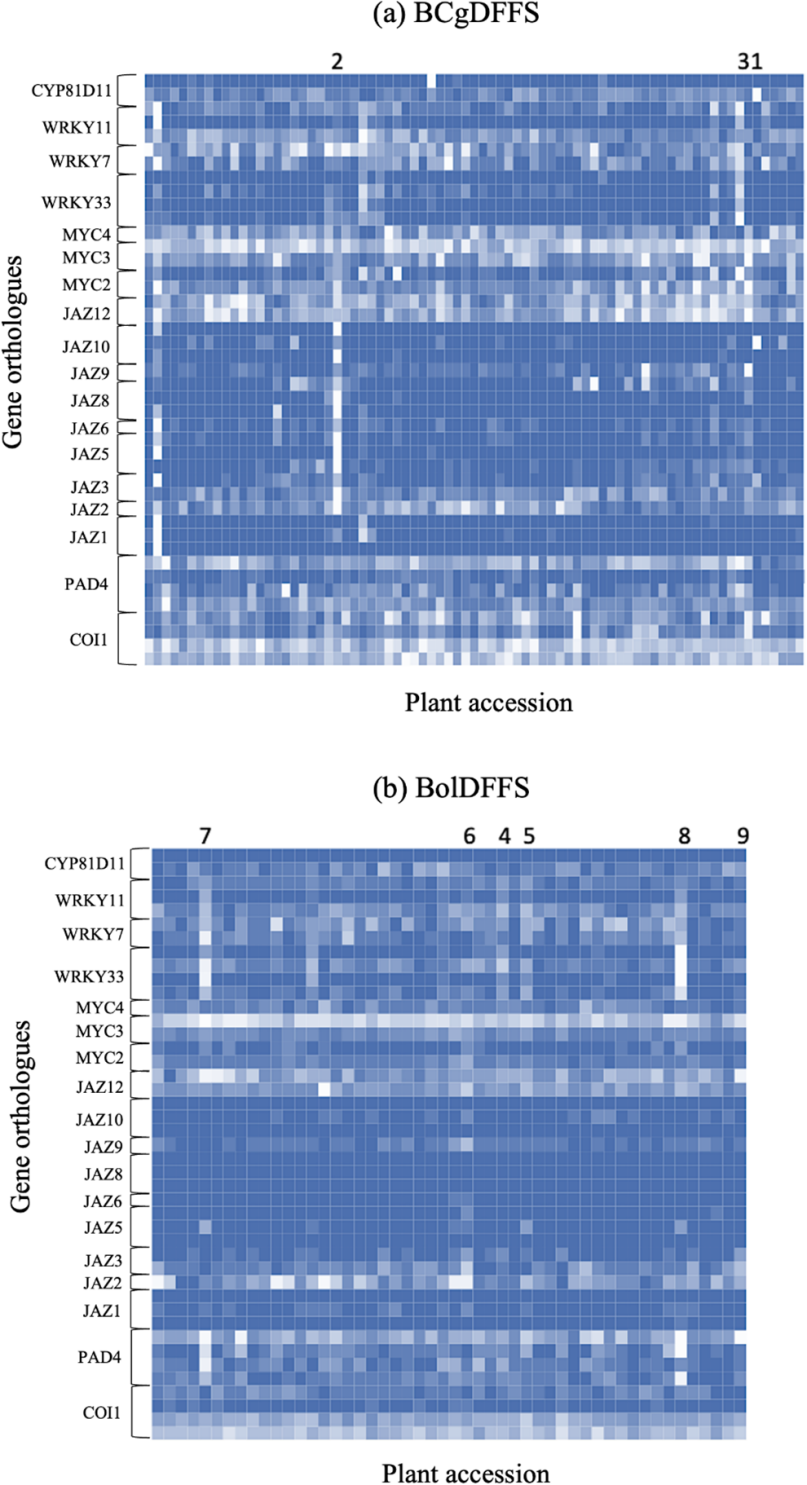
Heatmaps demonstrating relative pre‐infestation gene expression levels of *Brassica* jasmonic acid pathway transcription factors in (a) 77 *Brassica* accessions from BCgDFFS and (b) 50 *Brassica* accessions from BolDFFS. Lighter shades signify *Brassica* accessions with elevated levels of gene expression relative to other *Brassica* accessions evaluated. These heatmaps were used alongside calculated grand means of gene expression to guide the pre‐selection of a subset of *Brassica* accessions from each DFFS to screen for HPR to *Brevicoryne brassicae*. Heatmaps were generated using transcriptomic data (mRNA sequence reads) generated through the DEFRA‐funded vegetable genetic improvement network project (VeGIN, Teakle *et al*, data available upon request from https://warwick.ac.uk/fac/sci/lifesci/research/vegin/brassica/boldffs/). Columns topped with numbers identify the accessions screened for HPR to *B. brassicae*.

Grand means of mRNA sequencing reads across all 43 genes were calculated for the 77 C‐genome *Brassica* species accessions and the 50 *B. oleracea* accessions in the corresponding DFFS. Heatmaps of mRNA sequencing reads were also generated for both DFFS (Fig. 1). These heatmaps demonstrate in lighter shades the *Brassica* accessions with elevated levels of expression of each gene relative to the other *Brassica* accessions assessed. Heatmaps were used to visually assess the data and to assist in the selection of a subset of *Brassica* accessions for resistance screening alongside calculated grand means of expression data for each accession.

In BCgDFFS, the grand mean of mRNA reads across all 77 accessions ranged from 265.3 to 2065.9, with a mean average of 697.8. The accessions selected for resistance screen against *B. brassicae* [nos. 1, 2 and 3 – as indicated on Fig. 1(a)] were ranked 2^nd^, 3^rd^ and 6^th^, respectively, out of 77 accessions for the calculated grand mean. The accessions ranked 1^st^, 4^th^ and 5^th^ highest for the grand mean of gene expression were not selected for resistance screening owing to insufficient seed stocks. The grand mean of the selected accessions ranged from 1074.9 to 1812.6, with a mean average of 1467.6.

In BolDFFS, the grand mean of mRNA reads across all 50 accessions ranged from 301.3 to 1088.8, with a mean average of 523.5. The selected accessions [nos. 4, 5, 6, 7, 8 and 9 – as indicated on Fig. 1(b)] were ranked 7^th^, 5^th^, 2^nd^, 3^rd^, 1^st^ and 6^th^ (respectively) out of 50 accessions for the calculated grand mean. The accession ranked 4th highest for the grand mean of gene expression was excluded owing to insufficient seed stocks. The grand mean of the selected accessions ranged from 651.6 to 841.2, with a mean average of 841.2.

### 3.2 Antibiosis resistance screen

**Figure 2:**
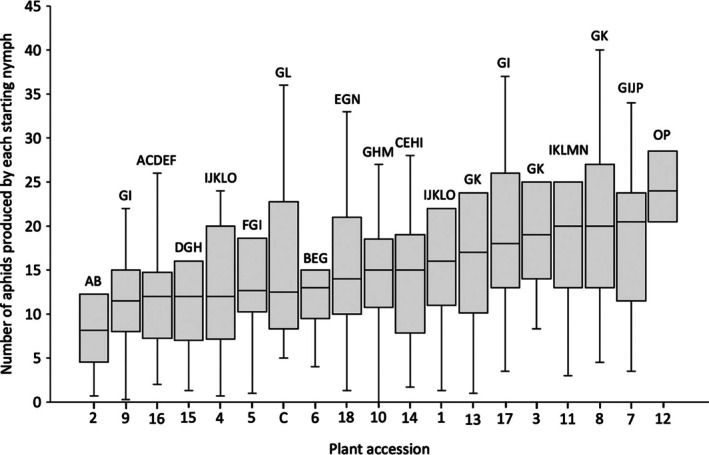
Antibiosis resistance screen results assessing population development of *Brevicoryne brassicae* on the 18 selected *Brassica* accessions and reference control (C) from an initial population of three 1‐day‐old nymphs. Accessions ranked according to the median number of aphids produced by each starting nymph. Plant accession was a significant factor influencing observed aphid numbers (*P* = 1.10 × 10^−9^). Between accessions, letters denote GLM *post hoc* test results, with accessions not sharing any letters significantly different in the number of *B. brassicae* found on them after the 2‐week screening period.

Following the 14‐day resistance screen, the median number of aphids produced by each of the three 1‐day‐old nymphs used to infest each plant ranged from 8 to 24 per accession (Fig. 2). There was a significant effect of plant accession on aphid population size [Wald χ^2^ (18473) = 79.370, *P* = 1.10 × 10^−9^]. The most resistant accession (*B. villosa*, accession no. 2) supported a significantly lower (*P* < 0.05) aphid population than 16 other accessions including the reference control. The most susceptible accession (*B. oleracea*, accession no. 12) supported a significantly larger (*P* < 0.05) aphid population than 15 other accessions including the reference control.

*Brassica* species had a significant effect on aphid population size [GLM, Wald χ^2^ (3473) = 31.132, *P* = 7.97 × 10^−7^], with *post hoc* testing revealing that *B. villosa* subsp. *tinei* (no. 2) and *Brassica cretica* (no. 16) both significantly reduced the number of aphids produced by each starting nymph relative to *B. oleracea* (*P* < 0.05), but there was no significant difference (*P* > 0.05) in aphid population size between *Brassica macrocarpa* and *B. oleracea*. *Brassica oleracea* cultivar group conversely did not exert a significant effect upon *B. brassicae* population size [Wald χ^2^ (6420) = 14.724, *P* = 0.023, failing to meet the Bonferroni‐correct significance threshold of 0.0125].

Further GLM analysis was undertaken to determine whether any of the different approaches used to select *Brassica* accessions for resistance screening performed significantly better in identifying resistant *Brassica* accessions. This analysis revealed that no selection method outperformed the others [Wald χ^2^ (4473) = 8.354, *P* = 0.079].

### 3.3 Aphid development rate and pre‐reproductive period

Nine Brassica accessions (including the reference control) were selected from the antibiosis screen for further studies on aphid development rate, reproduction and weight gain during development. The aim was to compare accessions that supported smaller populations of aphids (accession nos. 2, 4, 5, 9 and 16) against those supporting larger populations (accession nos. 11, 12 and 17).

**Figure 3:**
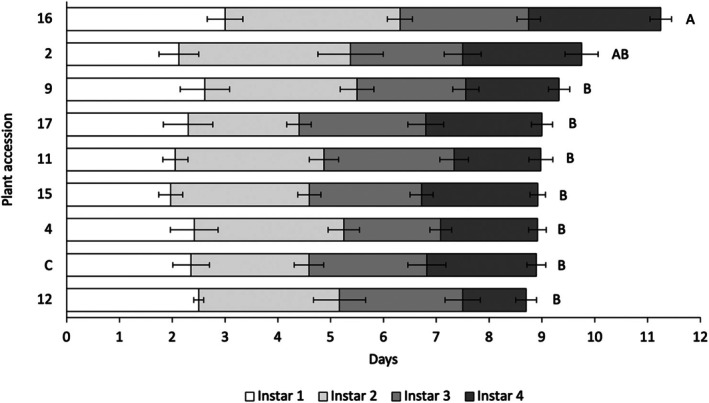
*Brevicoryne brassicae* instar development times and pre‐reproductive period on a sub‐set of eight *Brassica* accessions and the reference control (C). Total bar length corresponds to overall *B. brassicae* development time from birth to adulthood. Individual stacked bars correspond to duration of individual aphid instars. ANOVA revealed plant accession was a significant factor influencing overall aphid development time (*P* = 0.043). Accessions sharing no letters were found to significantly differ in their effect upon *B. brassicae* development time to adulthood (*P* = <0.05).

*Brevicoryne brassicae* time to adulthood across all accessions ranged from 4.5 to 14.5 days with a median of 9.5 days. The duration of instar phases were in the ranges 0.5–3.5 days for instar 1, 1–4 days for instar 2, 1–4 days for instar 3 and 0.5–4 days for instar 4, with medians of 1.5, 3.0, 2.0 and 2.0 days, respectively (Fig. 3).

ANOVA revealed a significant difference in overall development time of *B. brassicae* nymphs on different plant accessions [*F* (8, 65) = 2.095, *P* = 0.043]. Tukey's *post hoc* analysis revealed that plant accession no. 16 (*B. cretica*) significantly extended *B. brassicae* time to adulthood relative to all accessions apart from no. 2 (*B. villosa*). On plants of accession no. 16, newly born *B. brassicae* nymphs required an average of 11.25 days to reach adulthood compared to 8.9–9.75 days across all other accessions. Accession no. 16 thus extended total development time by a minimum of 1.5 days.

GLM analysis of each instar duration revealed that only instar 4 duration differed significantly between accessions [Wald *χ* ^2^ (8, 65) = 22.993, *P* = 0.003]. *Post hoc* analysis revealed that accessions nos. 2, 16 and 17, and the reference control significantly extended the duration of instar 4 relative to all other accessions. Instar 4 duration between accessions ranged from 1.2 to 2.5 days, with accession no. 16 offering the most significant slowing of this instar period and resulting in a duration double that of accession no. 12.

### 3.4 Aphid reproduction

**Figure 4:**
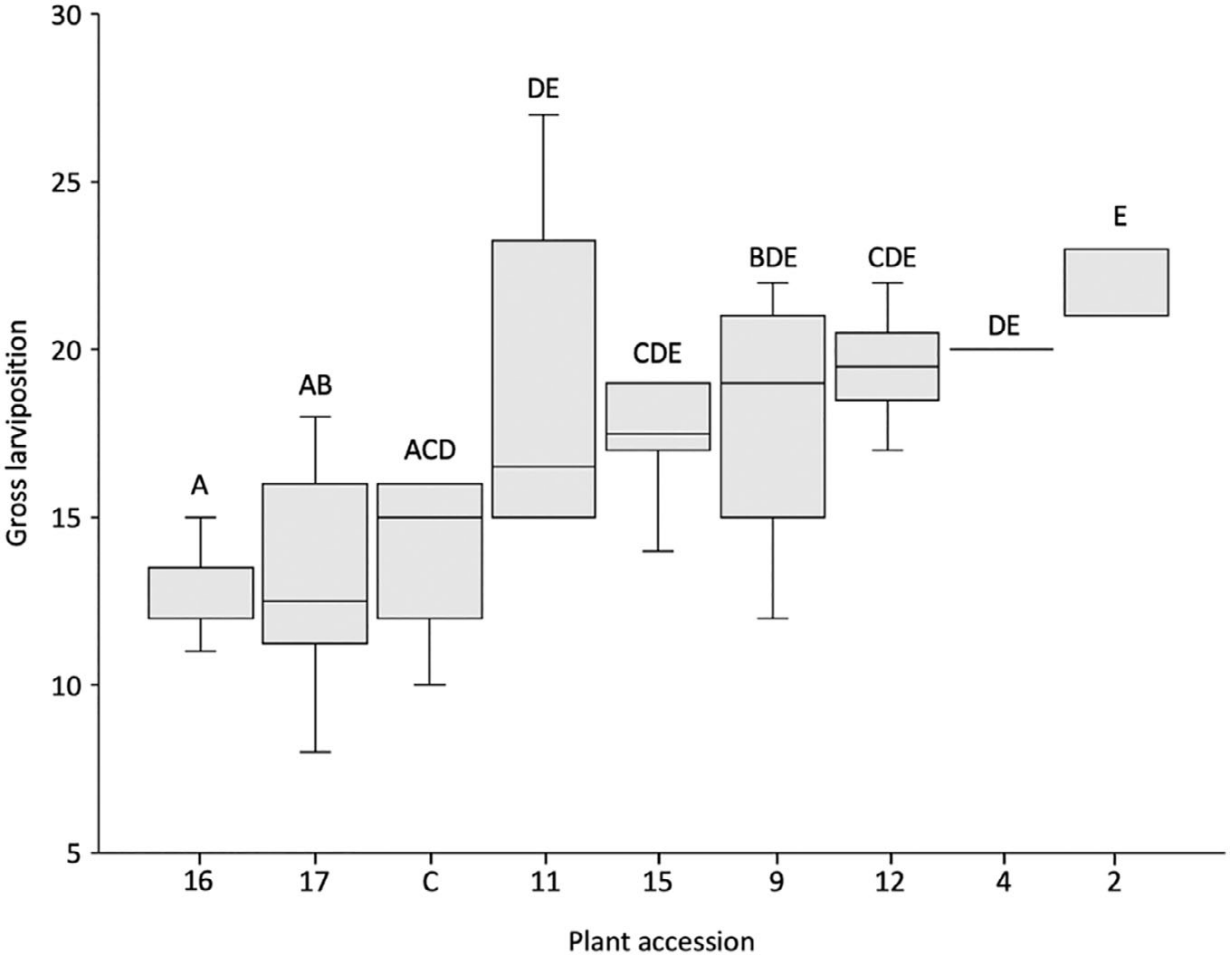
Gross larviposition of *Brevicoryne brassicae* over an 8‐day period when reared on selected *Brassica* accessions. GLM analysis revealed a significant difference in gross larviposition between accessions (*P* = 0.007). Accessions sharing no letters indicate a significant difference between *Brassica* accession in their effect upon gross *B*. brassicae larviposition as indicated by *post hoc* testing (*P* < 0.05).

Across all accessions, daily *B. brassicae* larviposition ranged from 0 to 14 nymphs, with a mean of 2.2. Gross larviposition during the observed period (equal to the pre‐reproductive period) ranged between accessions from eight to 37 nymphs, with a mean of 17.62 (Fig. 4). Separate GLM analyses revealed that plant accession had a significant effect upon both daily larviposition [Wald χ^2^ (8, 65) = 25.086, *P* = 0.002] and gross larviposition [Wald χ^2^ (8, 65) = 18.821, *P* = 0.016].

*Brassica cretica* accessions nos. 16 and 17 had the greatest effect upon daily and gross larviposition (Fig. 4). Both accessions reduced daily larviposition to a median of 2 (means of 1.5 and 1.8, respectively) and reduced gross larviposition to medians of 12.5 (mean of 12.8) and 13.0 (mean of 14.9), respectively. Accession no. 2 (*B. villosa* subsp. *tinei*) supported the largest amount of larviposition, with a median daily larviposition of 3 (mean of 2.5) and a median gross larviposition of 22 (mean of 23.2). *Post hoc* analyses revealed no significant differences in daily or gross larviposition between any of the evaluated *B. oleracea* accessions.

**Figure 5:**
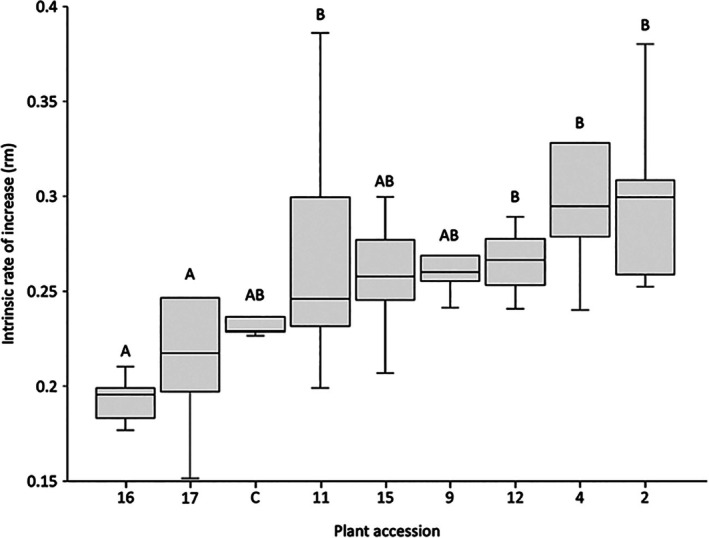
Intrinsic rate of increase of *Brevicoryne brassicae* reared on selected *Brassica* accessions. ANOVA revealed a significant different in intrinsic rate of increase between accessions (*P* = 4.70 × 10^−4^). Accessions sharing no letters indicate a significant difference in the effect of this accessions upon *B. brassicae* intrinsic rate of population increase indicated by *post hoc* ANOVA tests (*P* = <0.05).

**Figure 6:**
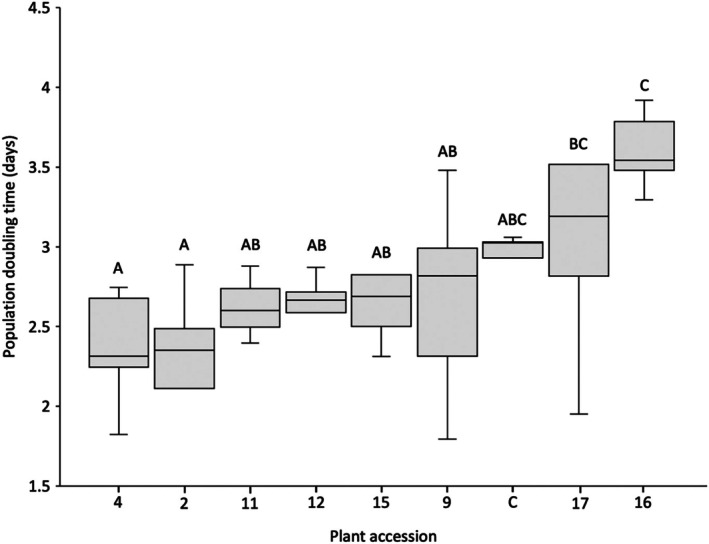
Population doubling time of *Brevicoryne brassicae* reared on selected *Brassica* accessions. ANOVA revealed a significant difference in population doubling time between accessions (*P* = 6.10 × 10^−5^). Accessions sharing no letters indicate a significant difference in the effect of this accessions upon *B. brassicae* population doubling time increase indicated by *post hoc* ANOVA tests (*P* = <0.05).

Intrinsic rate of population increase (*r*_m_) and population doubling time (*DT*) were calculated using gross larviposition results and aphid development times. Intrinsic rate of *B. brassicae* population increase across all accessions ranged from 0.15 to 0.39 with a mean of 0.26 (Fig. 5). Population doubling time across all accessions ranged from 1.79 to 4.58 days with a mean of 2.78 (Fig. 6). ANOVA revealed that plant accession had a significant effect on both intrinsic rate of population increase [*F* (8, 65) = 4.253, *P* = 4.70 × 10^−4^] and population doubling time [*F* (8, 65) = 5.253, *P* = 6.10 × 10^−5^].

*Post hoc* analyses revealed that *B. cretica* accession nos. 16 and 17 had the greatest limiting effect upon intrinsic rate of population increase, resulting in a mean *r*_m_ of 0.19 and 0.23, respectively – significantly lower than on accession nos. 2, 9, 11 and 4 (0.29, 0.27, 0.27 and 0.29, respectively). A similar trend was seen for population doubling time results, with *B. cretica* accession nos. 16 and 17 exhibiting an increased population doubling time to means of 3.6 and 3.2 days, respectively – significantly longer than population doubling times for *B. brassica* on accession nos. 4 (2.39 days) and 2 (2.39 days).

### 3.5 Aphid weight during development

**Figure 7:**
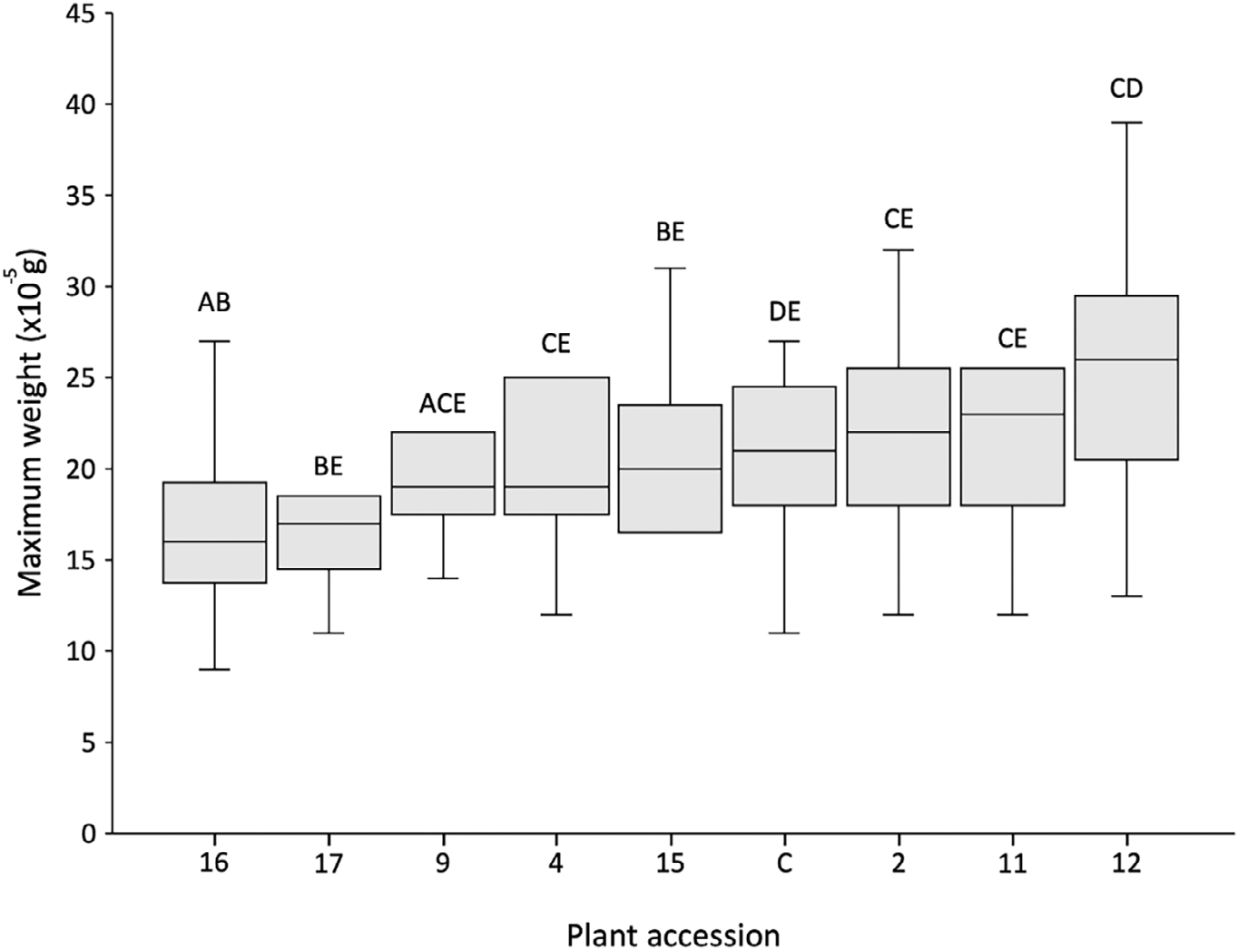
Maximum weight reached by *Brevicoryne brassicae* after 10 days on each evaluated *Brassica* accession, collating results from three aphid generations. ANOVA revealed a significant difference in maximum weight reached between *Brassica* accessions (*P* = 5.36 × 10^−7^). Accessions sharing no letters indicate a significant difference in maximum aphid weight reached between these accessions (*P* < 0.05).

**Figure 8:**
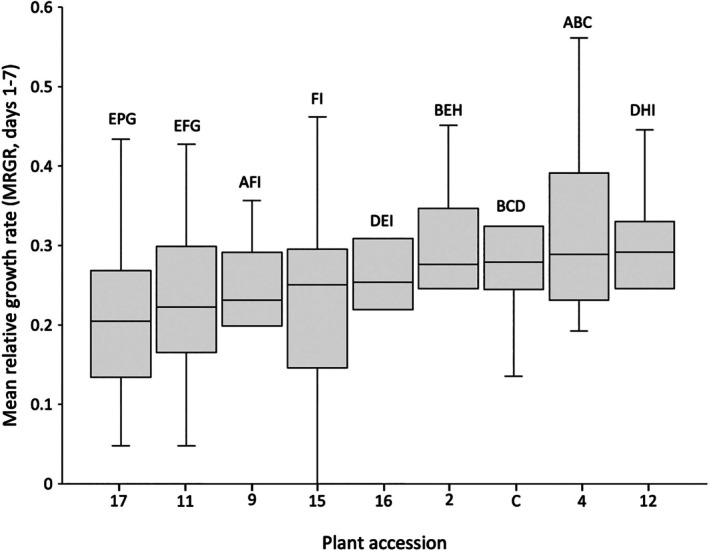
*Brevicoryne brassicae* mean relative growth rate (*MRGR*) between Day (D)1 and D7 following birth on different *Brassica* accessions. GLMM analysis revealed a significant difference in *B. brassicae MRGR* on different *Brassica* accessions (*P* = 1.82 × 10^−4^). Accessions sharing no letters indicate a significant difference in *B. brassicae MRGR* between these accessions (*P* < 0.05).

In order to evaluate *Brassica* accession effects upon aphid weight, two metrics were assessed over three *B. brassicae* generations on the same plants: (1) maximum aphid weight reached after 10 days (Fig. 7) and (2) mean relative growth rate between D1 and D7 (*MRGR*; Fig. 8). Statistical analyses revealed no significant differences between aphid generations for both maximum aphid weight reached [GLM, Wald χ^2^ (2,82) = 5.527, *P* = 0.063] and *MRGR* [GLMM, Wald χ^2^ (2,82) = 2.842, *P* = 0.241], thus maximum weight and *MRGR* growth results over the three generations were collated for subsequent analysis.

The GLM analysis revealed a significant difference in aphid weight reached when on different *Brassica* accessions [Wald χ^2^ (8,82) = 44.135, *P* = 5.36 × 10^−7^]. *Brassica cretica* accession nos. 16 and 17 exerted the greatest effect on maximum *B. brassicae* weight, limiting mean D10 weight to 16.4 × 10^−5^ g and 17.8 × 10^−5^ g, respectively (Fig. 7). *Post hoc* analyses confirmed that accessions No. 16 and No. 17 significantly limited maximum *B. brassicae* weight relative to accession nos. 11 and 12 (*P* < 0.05). Mean *B. brassicae* weight for all other accessions ranged from 19.9 to 25.1 × 10^−5^ g – with accession no. 12 resulting in the greatest average aphid weight.

Statistical analysis (GLMM) also confirmed a significant difference in *B. brassicae MRGR* (D1–D7) between *Brassica* accessions [*F* (8,82) = 4.004, *P* = 1.82 × 10^−4^]. Accession nos. 17, 11, 9 and 15 exerted the most notable limiting effect upon *MRGR*, with each resulting in *B. brassicae MRGR*s averaging 0.23 (Fig. 8). For all other accessions, *MRGR* ranged from 0.26 to 0.31, with *B. brassicae* on accession no. 4 demonstrating the highest observed *MRGR*. Mean weights of five aphids 1, 4, 7 and 10 days after birth over three generations on each *Brassica* accession are presented in Supporting information Table S1.

### 3.6 Aphid life‐history table and relationship between life parameters and initial partial resistance screening results

In order to summarize the results of antibiosis resistance screening and subsequent assays investigating HPR mechanisms, a life‐history table was produced (Table 3). Through stepwise linear regression analysis, development time to adulthood (pre‐reproductive period) was identified as the most significant explanatory variable of initial antibiosis screening results, accounting for 53.9% of variation observed in the initial antibiosis resistance screen when included as a sole explanatory variable [*F* (7, 10) = 8.177, *P* = 0.024, *R* ^*2*^ = 0.539]. The next most important explanatory variable was intrinsic rate of increase (*r*_m_), with its inclusion in the model accounting for a further 22.8% of variation in initial screening results when included as a second explanatory variable alongside ‘time to adulthood’ [*F* (6, 11) = 9.867, *P* = 0.013, *R* ^2^ = 0.767]. However, in this second linear regression model including both ‘total time to adulthood’ and ‘intrinsic rate of increase’ as explanatory variables, while ‘time to adulthood’ remained a significant factor (*P* = 0.004), ‘intrinsic rate of increase’ was no longer identified as a significant factor (*P* = 0.052). The first model including ‘development time to adulthood’ as a sole explanatory variable was therefore accepted.

**Table 3:** *Brevicoryne brassicae* life table summary when tested on nine different *Brassica* accessions

Metric		Statistic	*Brassica* accession								
			2	4	9	11	12	15	16	17	Reference Control
			*B. villosa*	*B. oleracea*	*B. oleracea*	*B. oleracea*	*B. oleracea*	*B. oleracea*	*B. cretica*	*B. cretica*	*B. oleracea*
Antibiosis screening results (aphids produced per starting nymph)		Mean	9.31	12.53	11.28	21.83	25.35	12.72	11.81	18.75	16.18
		Lower bound	6.23	9.26	8.72	16.09	20.27	9.62	9.43	14.98	13.19
		Upper bound	12.39	15.81	13.85	27.58	30.43	15.82	14.2	22.51	19.16
Aphid development (days)	Instar 1	Mean	2.13	2.42	2.62	2.06	2.5	1.97	3	2.3	2.36
		Lower bound	1.24	1.42	1.62	1.55	2.28	1.49	2.26	1.19	1.6
		Upper bound	3.01	3.41	3.61	2.56	2.72	2.46	3.74	3.41	3.11
		Median	1.5	1.5	1.5	1.5	2.5	1.5	2.75	1.5	2.25
	Instar 2	Mean	3.25	2.83	2.88	2.81	2.67	2.63	3.31	2.1	2.23
		Lower bound	1.78	2.18	2.21	2.22	1.4	2.15	2.81	1.57	1.62
		Upper bound	4.72	3.49	3.56	3.4	3.94	3.1	3.82	2.63	2.84
		Median	2.5	3	3	2.5	2.5	2	3	2	2
	Instar 3	Mean	2.13	1.83	2.06	2.47	2.33	2.13	2.44	2.4	2.23
		Lower bound	1.3	1.38	1.53	1.88	1.48	1.65	1.96	1.63	1.44
		Upper bound	2.95	2.29	2.59	3.05	3.19	2.6	2.91	3.17	3.02
		Median	2	2	2	3	2.5	2	2	2	2
	Instar 4	Mean	2.25	1.83	1.76	1.64	1.2	2.2	2.5	2.2	2.08
		Lower bound	1.51	1.47	1.34	1.16	0.64	1.89	2.06	1.75	1.69
		Upper bound	2.99	2.2	2.19	2.13	1.76	2.51	2.94	2.65	2.46
		Median	2	2	2	1.5	1	2	2	2	2
	Time to adulthood	Mean	9.75	8.92	9.32	8.24	7	8.38	11.25	9	8.43
		Lower bound	6.73	7.58	8.18	6.95	4.48	7.07	10.36	7.64	6.97
		Upper bound	12.77	10.26	10.46	9.52	9.52	9.69	12.14	10.36	9.88
		Median	8.5	8.5	9.5	8.5	8	8.5	11	9	9
Reproduction	Daily reproduction	Mean	2.68	2.5	2.18	2.38	2.44	2.26	1.6	1.86	1.9
		Lower bound	2.43	2.11	1.97	2.06	1.58	2.02	1.41	1.55	1.57
		Upper bound	2.94	2.89	2.39	2.69	3.3	2.51	1.8	2.18	2.23
		Median	3	2	2	2	2	2	2	2	2
	Gross reproduction	Mean	21.44	20	17.44	19	19.5	18.1	12.83	14.9	15.2
		Lower bound	18.42	17.37	14.57	14.88	16.19	15.78	11.29	8.88	9.03
		Upper bound	24.47	22.63	20.32	23.12	22.81	20.42	14.38	20.92	21.37
		Median	23	20	19	16.5	19.5	17.5	12.5	12.5	15
	Intrinsic rate of increase	Mean	0.29	0.29	0.27	0.27	0.26	0.26	0.19	0.23	0.25
		Lower bound	0.26	0.25	0.23	0.25	0.23	0.24	0.18	0.19	0.19
		Upper bound	0.33	0.34	0.32	0.28	0.3	0.28	0.21	0.27	0.31
	Population doubling time	Mean	2.39	2.39	2.66	2.62	2.64	2.7	3.6	3.22	2.82
		Lower bound	2.16	1.99	2.26	2.48	2.3	2.49	3.35	2.71	2.27
		Upper bound	2.62	2.79	3.06	2.76	2.98	2.91	3.85	3.73	3.36
Weight during development (× 10^−5^ g)	Maximum aphid weight (Day 10)	Mean	22.04	21.78	20.74	22.41	25.07	19.93	16.38	17.79	21.11
		Lower bound	19.87	19.17	18.25	20.19	22.45	17.39	14.51	15.5	19.46
		Upper bound	24.21	24.39	23.23	24.62	27.7	22.47	18.24	20.08	22.76
	Mean relative growth rate	Mean	0.3	0.32	0.24	0.23	0.28	0.23	0.25	0.22	0.29
		Lower bound	0.26	0.28	0.21	0.19	0.25	0.19	0.21	0.17	0.26
		Upper bound	0.33	0.37	0.27	0.27	0.31	0.28	0.29	0.26	0.32

The averages for each metric are reported with 95% confidence interval bounds.

## 4 DISCUSSION

In this controlled‐environment study significant differences were observed in the development of *B. brassicae* populations on different *Brassica* accessions, with a three‐fold difference in concluding aphid population between the most and least *B. brassicae*‐resistant accessions screened. While these findings require validation under field conditions, with different environmental conditions and crop development stage capable of exerting a significant effect upon the expression of HPR, such a difference in aphid population between *Brassica* accessions could have a significant effect upon the IPM strategy required for aphid management in field *Brassica* crops.

Accessions from four different *Brassica* species were screened – *B. cretica* Lam., *B. macrocarpa* Guss., *B. oleracea* and *B. villosa*. subsp. *tinei*. Lojac. In previous studies, several authors have identified that *Brassica* species can be a significant factor for resistance to insect pests.^47^, ^59^, ^60^ In our study, *B. cretica* and *B. villosa* demonstrated higher levels of partial HPR to *B. brassicae* relative to *B. oleracea* and *B. macrocarpa*. Accessions of *B. cretica* and *B. villosa* also resulted in the greatest effects on many assessed aspects of aphid developmental biology. Notably however, *B. cretica* accession no. 16 resulted in a significant extension of aphid pre‐reproductive period while *B. cretica* accession no. 17 did not – suggesting that even within species, effects of HPR upon different components of aphid biology may vary. Despite this, the results of this study identify *B. cretica*, particularly accession no. 16, as a promising source of potential HPR to *B. brassicae* – supporting previous research identifying *B. cretica* as a source of HPR to both *B. brassicae* and cabbage root fly, *Delia radicum* L. 1758.^46^, ^47^, ^61^

Introgression of (1) multigenic partial resistance and (2) resistance traits from related species both pose significant challenges to plant breeders owing to (a) challenges in identifying and tracking multiple minor‐effect genes in breeding programmes and (b) linkage drag of undesirable traits from related species, necessitating repeated rounds of backcrossing to restore agronomic viability. Nonetheless, both challenges have been overcome previously, leading to the successful development of pest‐resistant crop varieties. For example, introgression of partial HPR to carrot fly, *Chamaepsila rosae* Fabricius. identified in the variety ‘Sytan’ was achieved, permitting the release of two partially *C. rosae*‐resistant varieties to the amateur market, ‘Flyaway’ and ‘Resistafly’.^62^ Exploitation of HPR from a crop wild relative meanwhile was achieved during the development of lettuce varieties with *Nr1* HPR to currant‐lettuce aphid, *Nasonovia ribisnigri* – a trait introgressed from *Lactuca virosa* L.^15^

Recent genomic research has identified that *B. cretica* may be the closest extant relative of *B. oleracea*,^63^ thus introgression of minor‐gene traits from *B. cretica* into elite *B. oleracea* varieties may be feasible. Further research investigating HPR in a wider range of *B. cretica* accessions is therefore warranted. Such research should seek to determine the underlying mechanisms conferring HPR to *B. brassicae*, including whether resistance is conferred through molecular or phenotypic traits. While investigations of the molecular or physical mechanisms underlying *B. cretica* HPR were beyond the scope of the present study, a notable phenotypic trait of evaluated *B. cretica* accessions was glossy thick leaves even at a young development stage – a trait correlated in other studies with increased resistance to aphids.^64^

In addition to *Brassica* species, *B. oleracea* cultivar group was also evaluated in this study as a potential factor influencing HPR to *B. brassicae*. Owing to the application of a Bonferroni correction, no significant difference was observed between the six cultivar groups represented by the 12 *B. oleracea* accessions. However, considering (1) the small number of accessions representing each cultivar group, (2) the potential for false negative findings where Bonferroni corrections are applied, and (3) previous reports of significant differences between *B. oleracea* cultivar groups in their HPR to *B. brassicae*,^45^, ^47^ further investigation of cultivar group‐specific HPR to *B. brassicae* is warranted.

A final variable considered in relation to initial screening results was whether different approaches used to pre‐select *Brassica* accessions varied in their success at identifying varieties with HPR. Analysis revealed no significant difference in observed HPR to *B. brassicae* between (1) varieties previously reported in the literature as having partial HPR to *B. brassicae* and (2) varieties selected for screening from gene bank material with upregulated JA pathway transcription factor genes. It is thus unclear whether the latter novel pre‐selection approach proved successful. This approach was trialled to evaluate whether increasingly available transcriptomic datasets for gene bank material could form the basis of a high‐throughout pre‐selection approach for varieties to subsequently evaluate in resistance screens. Despite inconclusive results, such an approach warrants further evaluation, with advancements in understanding of the molecular mechanisms underlying HPR to aphid pests likely to provide better target genes to assess for upregulation as a basis for pre‐selection.

To date, the specific effects of resistant and partially resistant varieties upon horticultural pest development and reproduction have been notably understudied. Where evaluated, varieties with HPR have consistently been reported to elicit significant dampening of several aphid metrics including pest development rate, reproductive capacity and overall fitness across a range of pest species.^70^, ^73^ Significant HPR effects upon pest reproductive biology were observed in this study – with *Brassica* accessions identified as the most partially resistant to *B. brassicae* during initial screening also generally resulting in the most significant effects upon assessed *B. brassicae* metrics. Aphid pre‐reproductive period was identified as the most significant contributor to initial antibiosis resistance screening results, accounting for 53.9% of variation within the data. Previous studies where partial host plant resistance has been assessed in detail also have identified significant effects of HPR upon the duration of the aphid pre‐reproductive period.^70^, ^74^, ^75^ To the best of our knowledge, however, this study is the first to relate through linear regression analysis the results of an aphid population‐based HPR screen with the results of detailed assays evaluating HPR effects upon different aspects of aphid developmental biology. If a consistent feature of antibiosis HPR to aphids, knowledge that antibiosis resistance to aphids acts primarily by slowing aphid pre‐reproductive period could have significant implications for the integration of antibiosis HPR with other IPM tools. Further investigation in other crop species and with other aphid species is therefore warranted to confirm these findings.

While some accessions in this study such as no. 16 (*B. cretica*) demonstrated a significant dampening effect upon all evaluated *B. brassicae* metrics, most accessions demonstrated significant dampening effects upon only a subset of aphid metrics. This was the case for accession no. 17 (*B. cretica*). Alongside accession no. 16 (*B. cretica*), no. 17 also elicited among the most significant dampening of *B. brassicae* reproduction over time and maximum weight reached. Unlike accession no. 16, however, no. 17 did not elicit a significant dampening of aphid pre‐reproductive period – the metric subsequently identified as accounting for a majority of the variation in initial antibiosis resistance screening results. The inability of accession no. 17 to influence *B. brassicae* pre‐reproductive period is likely to have been reflected in its middling performance in the initial antibiosis resistance screen, despite otherwise significant dampening effects upon aphid biology. Conversely, despite the excellent performance of accession no. 2 (*B. villosa* subsp. *tinei*) in both the initial screen and in its ability to slow *B. brassicae* development to adulthood, subsequent assays revealed that *B. brassicae* on accession no. 2 demonstrated the highest recorded fecundity – undermining in part the HPR of this accession. Such variation between resistant plant varieties in their effects upon specific aspects of aphid reproductive biology have been noted previously.^12^, ^16^, ^73^ The findings of this study highlight that partial HPR to a specific pest may be a compound phenotype comprising independent (though potentially interactive) effects upon juvenile aphid development and adult reproduction. In this context, the results of this study highlight the importance of understanding the specific effects of varietal HPR upon pest biology, with the potential for both complementary and antagonistic effects upon overall HPR.

Within a conventional vegetable *Brassica* cropping system, a three‐fold difference in aphid population development throughout the season, as identified in this study, could significantly reduce the frequency and number of chemical pesticide applications required to manage aphid populations below economically damaging levels. Such a reduction in aphid numbers may permit aphid control on conventional farms without the need to resort to broad‐spectrum PPP such as pyrethroids. However, beyond simply reducing the required frequency of PPP application, studies have demonstrated further benefits of combining HPR with insecticides, particularly where selective chemistry is used. One such study demonstrated that combined use of the selective insecticide chlorantraniliprole and tomato varieties with HPR to *Tuta absoluta* resulted in significantly improved control on a per‐application basis relative to use of chlorantraniliprole on a susceptible tomato variety.^65^ The deployment of partial HPR may therefore permit both increased efficacy and reduced required frequency of chemical insecticides applications – limiting harmful adverse effects upon released and naturally occurring populations of aphid predators on farms.

As demonstrated in the present study, HPR can significantly influence initial aphid infestation, reduce the rate of pest population development and slow the speed of pest spread through a crop. Through these effects upon aphid biology partial HPR can serve as a foundational tool within IPM strategies, with significant scope for beneficial interactions with other components of an IPM strategy including biopesticides and biological controls.^4^, ^6^, ^66^, ^67^, ^68^ For example, increasing the duration of the *B. brassicae* pre‐reproductive period and thus extending the duration of individual aphid nymph instar phases could have a significant positive effect upon the efficacy of contact‐acting biopesticides with a delayed mode‐of‐action such as entomopathogenic fungi (EPF).^69^ This was demonstrated by Gladman (2022), who identified significantly increased aphid nymph mortality when equal doses of the entomopathogenic fungus *Akanthomyces dipterigenus* were applied to fixed age aphid nymphs on partially‐resistant *Brassica* varieties relative to more susceptible varieties – with partial HPR therefore acting as a potentiator for fungal entomopathogens.^69^ Host plant resistance also may interact with biological controls including parasitoid wasps – with several authors noting that parasitoid survival and rates of aphid parasitism varies significantly dependent upon relative levels of HPR.^70^, ^71^ The nature of this interaction, however, is uncertain, with different authors noting either synergistic or antagonistic effects between HPR and parasitoids dependent upon the plant and aphid species evaluated.^70^, ^71^ Despite these studies, interactions between HPR and other biological components of IPM strategies remains a significant knowledge gap.^6^

Knowledge and understanding of interactions between HPR and other IPM tools is vital for maximizing the efficacy and long‐term management of partial HPR. Previously where single‐gene HPR to insect pests has been developed and released commercially, it has most commonly been deployed in isolation as a direct substitute for chemical insecticides.^14^ Such an approach, however, places significant selection pressure upon pest populations, leading to erosion or complete breakdown of HPR.^14^, ^72^ This has been typified by the biotypic breakdown of *Nr1* resistance to *N. ribisnigri* in previously resistant lettuce varieties through the emergence of the *Nr1‐insensitive Nr2 N. ribisnigri* biotype.^15^ To date, owing to a relative absence of commercial varieties developed with partial HPR to invertebrate pests, the long‐term in‐field resilience of partial HPR to significant pest pressure is uncertain. Nonetheless, case studies with single‐gene HPR highlight the importance of effective and strategic management of HPR. This is likely to be best achieved through deploying partially resistant varieties as one component of a larger IPM strategy. Such an approach should increase the durability of partial HPR, placing as it does a range of competing selection pressures upon insect pests, akin to rotating pesticide modes of action.

## 5 CONCLUSIONS

With mounting pressure on growers to reduce use of synthetic chemical pesticides, the development of successful and sustainable IPM strategies for aphid control in field crops is paramount. While it is clear that partial HPR will not, on its own, provide sufficient aphid control to satisfy growers and meet stringent horticultural quality thresholds, the results of this study demonstrate the significant effects that partial HPR can exert upon *B. brassicae* population development and reproductive biology. In particular, aphid pre‐reproductive was identified as the aphid parameter most significantly perturbed by partial HPR. Such HPR‐mediated effects have the potential to significantly influence and interact with other components of IPM strategies. Further research is warranted to characterize the specific effects of partial HPR upon aphid pests and to investigate the subsequent effects of these perturbations upon other components of IPM.

## FUNDING INFORMATION

This research was funded by the Biotechnology and Biological Sciences Research Council (BBSRC) through the Midlands Integrated Biosciences PhD Training Programme (MIBTP). Project reference: 1897825. Understanding interactions between biopesticides and partial crop resistance for management of aphid pests of brassica crops.

## CONFLICT OF INTEREST

The authors declare no conflict of interest. The funder had no role in the design of the study; in the collection, analyses, or interpretation of data; in the writing of the manuscript, or in the decision to publish the results.

## AUTHOR CONTRIBUTIONS

A.K.G, D.C., and G.P. designed the experiments. A.K.G. performed all experiments, analysed the data, and wrote the paper. A.K.G. and G.T. undertook the analyses to select plant accessions for screening. A.K.G. was awarded the PhD studentship with D.C. and G.T. which funded this research. All authors have read and agreed to the published version of the manuscript.

## Supporting information


**Supplementary Table S1.** Weight of five *Brevicoryne brassicae* 1, 4, 7 and 10 days after birth over three generations on different *Brassica* accessions.

## Data Availability

The data that support the findings of this study are available from the corresponding author upon reasonable request.
