# Supplementary material for: Evaluating antibiosis resistance to cabbage aphid (Brevicoryne brassicae L., 1758) in vegetable brassicas (Brassica oleracea L.) and related C‐genome brassica species
Source: Pest Manag Sci. 2025 Aug 29;81(12):8431–47. doi: 10.1002/ps.70161 (PMC12618919; doi:10.1002/ps.70161)
Supplement: Supplementary file 1 — Supplementary Table S1. Weight of five Brevicoryne brassicae 1, 4, 7 and 10 days after birth over three generations on different Brassica accessions. [file PS-81-8431-s001.docx]

| ***Brassica* accession** | **Weight of five aphids (x 10^-5^g) ± 95% confidence interval** | | | |
| --- | --- | --- | --- | --- |
|  | **Day 1** | **Day 4** | **Day 7** | **Day 10** |
| ***Brevicoryne brassicae* generation 1** | | | | |
| 2 | 2.67 ± 0.65 | 6.44 ± 1.57 | 14.33 ± 2.88 | 17.89 ± 2.48 |
| 4 | 2.67 ± 1.13 | 10.44 ± 1.14 | 21.11 ± 2.48 | 27.00 ± 5.41 |
| 9 | 3.33 ± 1.18 | 6.11 ± 1.89 | 12.11 ± 3.75 | 24.44 ± 6.21 |
| 11 | 4.22 ± 1.07 | 7.00 ± 2.29 | 13.44 ± 4.93 | 22.22 ± 2.77 |
| 12 | 3.56 ± 1.43 | 7.33 ± 2.12 | 14.78 ± 3.96 | 26.89 ± 5.00 |
| 15 | 4.22 ± 1.26 | 7.22 ± 1.02 | 14.00 ± 2.57 | 20.67 ± 4.07 |
| 16 | 2.67 ± 0.73 | 4.33 ± 1.27 | 11.89 ± 2.97 | 18.33 ± 2.07 |
| 17 | 2.56 ± 0.47 | 5.44 ± 1.54 | 12.56 ± 5.30 | 18.67 ± 4.14 |
| Doric | 3.11 ± 0.76 | 6.89 ± 2.37 | 15.11 ± 2.72 | 21.67 ± 3.61 |
| ***Brevicoryne brassicae* generation 2** | | | | |
| 2 | 2.89 ± 0.83 | 6.00 ± 1.13 | 17.44 ± 2.87 | 21.33 ± 2.49 |
| 4 | 2.56 ± 0.74 | 7.33 ± 1.50 | 14.33 ± 2.77 | 19.67 ± 3.06 |
| 9 | 3.22 ± 0.85 | 5.33 ± 1.18 | 14.00 ± 1.96 | 19.00 ± 2.24 |
| 11 | 3.56 ± 0.87 | 6.89 ± 1.20 | 15.22 ± 1.95 | 21.44 ± 5.15 |
| 12 | 3.11 ± 0.83 | 6.89 ± 1.77 | 20.11 ± 3.02 | 24.56 ± 2.81 |
| 15 | 4.00 ± 0.73 | 6.56 ± 0.74 | 15.00 ± 3.95 | 19.33 ± 5.70 |
| 16 | 2.44 ± 0.47 | 5.44 ± 2.17 | 9.56 ± 2.19 | 15.44 ± 3.83 |
| 17 | 3.33 ± 0.73 | 6.11 ± 2.08 | 10.33 ± 2.33 | 17.56 ± 3.46 |
| Doric | 3.22 ± 0.29 | 8.11 ± 1.44 | 17.22 ± 1.84 | 21.00 ± 2.55 |
| ***Brevicoryne brassicae* generation 3** | | | | |
| 2 | 3.67 ± 1.08 | 7.78 ± 1.38 | 20.00 ± 3.20 | 26.89 ± 3.11 |
| 4 | 2.78 ± 0.63 | 6.22 ± 1.52 | 16.33 ± 3.47 | 18.67 ± 1.39 |
| 9 | 2.78 ± 0.71 | 6.33 ± 1.27 | 11.67 ± 2.01 | 18.78 ± 1.26 |
| 11 | 3.22 ± 0.78 | 8.78 ± 1.95 | 15.78 ± 5.10 | 23.56 ± 2.89 |
| 12 | 3.44 ± 0.74 | 9.11 ± 3.11 | 17.22 ± 2.57 | 23.78 ± 5.06 |
| 15 | 2.44 ± 0.81 | 5.89 ± 1.74 | 11.78 ± 2.58 | 19.78 ± 2.79 |
| 16 | 2.5 ± 0.36 | 7.00 ± 0.58 | 14.5 ± 1.88 | 14.83 ± 1.82 |
| 17 | 3.5 ± 0.36 | 9.67 ± 2.25 | 14.00 ± 1.94 | 16.83 ± 3.16 |
| Doric | 2.56 ± 0.66 | 8.56 ± 2.34 | 16.11 ± 2.54 | 20.67 ± 2.07 |

**Supp. Table S1: Weight of five *Brevicoryne brassicae* 1, 4, 7, and 10 days after birth over three generations on different *Brassica* accessions**
